# Antimicrobial, Anti-inflammatory, Angiogenesis, and Wound Healing Activities of Copper Nanoparticles Green Synthesized by *Lupinus arcticus* Extract

**DOI:** 10.5812/ijpr-147434

**Published:** 2025-03-26

**Authors:** Nizar H. Saeedi, Abdullah D. Alanazi, Rawaf Alenazy, Abdullah F. Shater

**Affiliations:** 1 Department of Medical Laboratory Technology, Faculty of Applied Medical Sciences, University of Tabuk, Tabuk 71491, Saudi Arabia; 2 Department of Biological Sciences, Faculty of Science and Humanities, Shaqra University, Ad-Dawadimi 11911, Saudi Arabia; 3 Department of Medical Laboratory, College of Applied Medical Sciences-Shaqra, Shaqra University, Shaqra 11961, Saudi Arabia

**Keywords:** Drug Delivery, Biofilm, Wound Healing, Green Synthesis

## Abstract

**Background:**

Wound healing and antibiotic resistance of pathogenic microbes have become global issues with serious consequences for the treatment of infectious diseases.

**Objectives:**

The present study aimed to evaluate the antibacterial, anti-inflammatory, angiogenic, and wound healing properties of copper nanoparticles (CuNPs) synthesized using *Lupinus arcticus* extract.

**Methods:**

The green synthesis was conducted using the precipitation method. The antibacterial activity of CuNPs against both methicillin-sensitive and methicillin-resistant *Staphylococcus aureus* (MRSA) strains was evaluated. The effects of CuNPs on protein leakage, the expression levels of biofilm-related genes [e.g., intracellular adhesion A (icaA), intracellular adhesion D (icaD), and elastin-binding protein (EbpS) genes] in MRSA, as well as its impact on wound healing, angiogenesis, and anti-inflammatory effects, were assessed.

**Results:**

The CuNPs exhibited a spherical shape with dimensions ranging from 10 to 85 nm. Both CuNPs alone and in combination with gentamicin (GNT) inhibited biofilm formation in MRSA, with minimum biofilm inhibitory concentration (MBIC_50_) values of 6.6 µg/mL and 0.50 µg/mL for MRSA, respectively. The CuNPs significantly (P < 0.05) downregulated the expression levels of icaA, icaD, and EbpS in MRSA, particularly at half the minimum inhibitory concentration (1/2 MIC) and the minimum inhibitory concentration (MIC). Additionally, CuNPs markedly (P < 0.001) increased protein leakage in MRSA. The CuNPs demonstrated potent in vitro wound healing effects, promoting fibroblast cell proliferation and wound closure in a dose-dependent manner. Our results indicated a significant (P < 0.05) increase in the expression of HLA-G5 and VEGF-A genes in cells exposed to CuNPs. Furthermore, CuNPs reduced the expression levels of inflammatory genes in lipopolysaccharide (LPS)-stimulated RAW 264.7 cells (P < 0.05).

**Conclusions:**

The findings of this experimental test indicate that CuNPs, particularly in conjunction with GNT, exhibits promising antibacterial effects against MRSA without causing cytotoxicity to normal cells. This study also demonstrated that green-synthesized CuNPs possesses significant wound-healing properties through its antibacterial activity, inhibition of biofilm formation, induction of angiogenesis, and reduction of inflammation. However, further experiments are necessary to elucidate the precise mechanisms of action and potential toxicity of CuNPs.

## 1. Background

*Staphylococcus aureus* is an anaerobic bacterium and is considered the most significant species within the genus *Staphylococcus* (1). As the most prevalent bacterial agent, it is responsible for over 70% of soft tissue infections, leading to a range of clinical symptoms, from skin infections such as boils and abscesses to severe conditions like pneumonia, osteomyelitis, toxic shock syndrome, meningitis, and septicemia (2). Currently, antibiotic resistance among pathogenic microbes has emerged as a global issue with serious implications for the treatment of infectious diseases. This resistance results in a diminished effectiveness of antibiotics in managing these conditions (3). The emergence of antibiotic resistance in strains of *S. aureus* is attributed to the acquisition of multiple resistance factors (4). Biofilms are structured communities of bacteria that adhere to surfaces and are composed of various substances, including extracellular polymers, exopolysaccharides, nucleic acids, and proteins (5). Furthermore, the formation of biofilms is a critical mechanism that hinders the effectiveness of antibiotics in treating staphylococcal infections (6). It is now well-established that wounds resulting from conditions such as diabetes, gastric disorders, and duodenal ulcers significantly affect individuals’ quality of life (7). The process of wound healing is complex, involving phases of inflammation, angiogenesis, cellular proliferation, and tissue remodeling, all characterized by interactions between cells and the extracellular matrix (8). In recent years, a novel approach to managing drug-resistant hospital infections and enhancing wound healing has emerged: The application of nanotechnology in the medical field (9). Today, nanoparticles (NPs) offer several advantages, including high bioavailability, improved solubility of hydrophobic compounds, enhanced pharmacokinetics of active pharmaceutical ingredients, and low toxicity, making them valuable for medical and pharmaceutical applications (10). Metal NPs, distinguished by their unique physical properties, are frequently employed as a promising option among various types of NPs for delivering both small drug molecules and large biomolecules (11).

Currently, there is a significant focus on the production of NPs through biosynthesis using natural agents such as plants, bacteria, and fungi (12). Recent studies have highlighted the use of herbs and their derivatives, commonly referred to as "green synthesis", as a cost-efficient, environmentally friendly, and reliable method for producing NPs (13). Copper exhibits significant antimicrobial properties against a diverse array of microorganisms, and this efficacy is notably enhanced when copper is appropriately nano-dispersed (14). In numerous investigations concerning metal-based nano-antimicrobials, it has been observed that the biological efficacy of these materials is markedly superior or prolonged in comparison to the established bioactivity of the corresponding bulk metal (14). Several studies have reported the antibacterial effects of various metal NPs against both gram-negative and gram-positive bacteria (14). Copper nanoparticles (CuNPs) exhibit several therapeutic activities, including anticancer, antioxidant, antinociceptive, and anti-inflammatory properties (15). Additionally, CuNPs demonstrated antimicrobial effects against both positive and negative bacteria (e.g., *Bacillus* spp., *Salmonella* spp., and *Staphylococcus* spp.), pathogenic fungi (e.g., *Candida* spp., *Aspergillus* spp., and *Fusarium *spp.), as well as viral strains (e.g., human influenza A and avian influenza) and parasitic organisms (e.g., *Leishmania* spp., *Toxoplasma gondii*, and *Echinococcus* spp.) (16).

## 2. Objectives

The present study aimed to measure the antibacterial, anti-inflammatory, angiogenesis, and wound healing activities of CNP green synthesized using *Lupinus arcticus* extract against *S. aureus* resistant to methicillin strain.

## 3. Methods

### 3.1. Synthesis of Copper Nanoparticles

#### 3.1.1. Plant Extract

The aerial components of *L. arcticus* were obtained from a botanical marketplace in Riyadh, Saudi Arabia, in June 2022. After identification, the plant was assigned voucher number 52-2022 and preserved at Shaqra University in Saudi Arabia. Subsequently, 250 grams of the plant material were extracted using water for 72 hours at 21°C, employing the percolation technique.

#### 3.1.2. Green Synthesis

The eco-friendly synthesis was performed using a precipitation technique. An aqueous extract (0.05 L) was added to a beaker containing a copper sulfate solution (0.1 L, 1 mM) and stirred for 10 minutes. The mixture was then maintained at 24°C for 12 hours. The change in color of the solution to a dark yellow hue, along with the emergence of turbidity, indicated the successful synthesis of CuNPs (17).

#### 3.1.3. UV-Vis Spectroscopy Analysis

To investigate the transformation of copper ions into CuNPs, a solution containing NPs (0.3 mL) was combined with 3 mL of normal saline. This mixture was then analyzed using UV-visible spectroscopy with a Shimadzu UV-2550 spectrophotometer (Japan) over a wavelength range of 300 - 700 nm.

#### 3.1.4. Physical Characterization of Copper Nanoparticles

The dimensions and morphology of the CuNPs produced via a green synthesis method were evaluated using a scanning electron microscope (SEM) with specific parameters, including a magnification of 10x, a resolution of 1 nm, and an operating voltage of 15 kV. Additionally, dynamic light scattering (DLS) equipment from Malvern, UK, known as the Zeta Sizer, was employed for further analysis.

#### 3.1.5. X-ray Diffraction Analysis

The study evaluated the incorporation of copper in CuNPs and its crystal structure using X-ray diffraction (XRD) analysis. This assessment was conducted using a copper lamp as the X-ray source, which emitted beams with a wavelength of λ = 1.54 Å, and an XRD device, model 2000 APD, from Italy.

#### 3.1.6. Fourier Transform Infrared Spectroscopy Analysis

The fourier transform infrared spectroscopy (FTIR) analysis was performed to identify the biomolecules that act as coating agents for the synthesized NPs. In summary, the CuNPs powder was mixed with potassium bromide to create tablets, which were subsequently analyzed using a Tensor 27 device from Germany.

### 3.2. Antibacterial Properties of Copper Nanoparticles

The methicillin-resistant *Staphylococcus aureus* (MRSA) strain (ATCC 33591) was obtained from Shaqra University in Saudi Arabia and was cultured on Mueller-Hinton agar (MHA) and Mueller-Hinton broth (MHB) (Heraeus, Germany) at 37°C in a 5% CO_2_ environment.

#### 3.2.1. The Standard McFarland 0.5 Solution

The solution was formulated by prior research by blending bacterial colonies with physiological serum and assessing the turbidity of the mixture in relation to the dilution of a standard 0.5 McFarland solution (18).

#### 3.2.2. Antibacterial Effects of Copper Nanoparticles

The antibacterial properties of CuNPs were investigated following the guidelines established by the Clinical and Laboratory Standards Institute (CLSI). This was accomplished by determining the minimum inhibitory concentration (MIC) and minimum bactericidal concentration (MBC) using a microdilution assay in 96-well microtiter plates. To summarize, 0.05 mL of MHB was dispensed into all wells except the control wells. Subsequently, 100 µL of the synthesized CuNPs were added to the first two rows (control wells), with serial dilutions performed from the second row to the tenth row. This process involved transferring 50 µL from the second row to the third row, and so forth, up to the tenth row. Following this, 50 µL of a 24-hour microbial culture, equivalent to half the turbidity of a McFarland standard of 1.5 × 10^8^ CFU/mL (measured at an absorbance of 625 nm), were added to rows two through ten. The plates were then incubated at 37°C for 24 hours. After incubation, 2,3,5-triphenyltetrazolium chloride (TTC) was introduced to the wells. Wells that remained colorless were identified as the MIC. Subsequently, wells that lacked color were streaked onto MHA plates, and the lowest concentration without visible colonies was determined as the MBC.

### 3.3. Inhibition of Biofilm Production

The study evaluated the effects of CuNPs on the inhibition of biofilm formation in MRSA using a previously described method. In summary, a bacterial suspension was mixed with varying concentrations of CuNPs in a 96-well microtiter plate and incubated for 24 hours at 37°C. Positive controls consisted of bacterial suspensions without CuNPs, while negative controls included wells containing brain-heart infusion broth (BHIB) with 2% sucrose. After washing and removing free cells, the crystal violet assay was employed to assess cell adhesion and biofilm formation. The absorbance of the wells was measured at 600 nm using a microplate reader. The minimum biofilm inhibition concentration (MBIC_50_) was determined as the lowest concentration of CuNPs that inhibited biofilm production by at least 50%.

### 3.4. Effect on the Expression Level of Biofilm Genes

The effects of CuNPs on the expression levels of biofilm-related genes [e.g., intracellular adhesion A (icaA), intracellular adhesion D (icaD), and elastin-binding protein (Ebps) genes] of MRSA were studied by quantitative real-time PCR. Briefly, the bacterial suspension was exposed to CuNPs at 1/2 MIC and MIC at 37°C for 24 hours. After extracting the total RNA using a commercial kit (Qiagen, Germany), complementary DNA (cDNA) was synthesized based on the cDNA reverse transcription kit (Qiagen, Iran). The effect of CuNPs on the expression level of icaA, icaD, and EbpS genes was studied by SYBR Green real-time PCR. The primers of these genes and gyrB as the housekeeping gene are shown in Table 1. The thermal reaction was planned as denaturation for 15 minutes at 96°C, annealing for 20 seconds at 57°C, and extension for 25 seconds at 72°C in 40 cycles. The expression level was measured by the 2^−△△CT^ technique using iQTM5 optical system software (Bio-Rad, Hercules, CA) (19).

**Table 1:** . The List of the Primers Used in This Study

Primers and Sequence (5′→3′)	Size (bp)
**icaA**	188
F: ACACTTGCTGGCGCAGTCAA	
R: TCTGGAACCAACATCCAACA	
**icaD**	198
F: ATGGTCAAGCCCAGACAGAG	
R: AGTATTTTCAATGTTTAAAGCAA	
**EbpS**	180
F: CATCCAGAACCAATCGAAGAC	
R: CTTAACAGTTACATCATCATGTTTATC	
**VEGF-A**	173
F: AGGGCAGAATCATCACGAAGT	
R: AGGGTCTCGATTGGATGGCA	
**HLA-G5**	185
F: CTGAGATGGAAGCAGTCTT	
R: GCTCCCTCCTTTTCAATCT	
**TNF-α**	205
F: AGTTCCCAAATGGCCTCCCTCTCA	
R: GTGGTTTGCTACGACGTGGGCT	
**IL-1β**	89
F: GCAACTGTTCCTGAACTCAACT	
R: ATCTTTTGGGGTCCGTCAACT	
**NF-κB**	169
F: GCGGGAGAGGGGATTCCCTGCGGCCCCG	
R: CGGGGCCGCAGGGAATCCCCTCTCCCGC	
**GAPDH**	223
F: AACTTTGGCATTGTGGAAGG	
R: ACACATTGGGGGTAGGAACA	

Abbreviations: icaA, intracellular adhesion A; icaD, intracellular adhesion D; EbpS, elastin-binding protein; VEGF-A, vascular endothelial growth factor; HLA-G5, human leukocyte antigen-G5; TNF-α, tumor necrosis factor alpha; IL-1β, interleukin-1; NF-κB, nuclear factor kappa B.

### 3.5. Effect of Copper Nanoparticles on Protein Leakage

In summary, a bacterial solution was exposed to varying concentrations of CuNPs at 1/4 MIC, 1/3 MIC, and 1/2 MIC at 37°C for 2 hours. Subsequently, the suspension was centrifuged at 4,000 revolutions per minute for 5 minutes. Following centrifugation, 950 μL of Bradford’s reagent was added to assess the protein content using the Bradford method. Positive and negative controls were established with sodium dodecyl sulfate (SDS) and normal saline, respectively. The absorbance was measured at 590 nm using a microplate reader spectrophotometer (20).

### 3.6. Wound Healing Effects of Copper Nanoparticles

#### 3.6.1. Cell Culture

The human skin fibroblast (Hs27) and macrophage (RAW 264.7) cell lines were procured from the American type culture collection (ATCC) and maintained in DMEM supplemented with 10% FBS and 1% penicillin-streptomycin at 37°C in a 5% CO_2_ incubator. The cell density was subsequently standardized to 1 × 10^5^/mL utilizing a hemocytometer.

#### 3.6.2. Cell Viability Assay

The MTT assay was employed to investigate the effect of CuNPs on cell viability (21). Briefly, a cell concentration of 1,000,000 cells/mL was exposed to CuNPs concentrations ranging from 25 to 200 µg/mL in 96-well plates for 72 hours at 37°C. Subsequently, MTT solution (5 mg/mL) from Sigma-Aldrich, Germany, was added to the wells and incubated under the same conditions. After adding dimethyl sulfoxide, the optical density (OD) was measured at 570 nm using an ELISA reader (LX800; Biotec, USA). Cytotoxicity was assessed by determining the cytotoxic concentration that affects 50% of the cells (CC_50_).

#### 3.6.3. Fibroblast Proliferation Assay

The fibroblast proliferation assay was conducted by introducing 0.1 mL of fibroblast cells (1 × 10^5^ cells/mL) into individual wells of a 96-well plate and allowing them to incubate for 24 hours. After removing the supernatant, CuNPs and asiaticoside (used as a positive control) were added to the wells, followed by an additional 24-hour incubation. Subsequently, MTT solution (5 mg/mL) was added to the wells and incubated in a 5% CO_2_ environment at 37°C for 4 hours. The absorbance was measured at 570 nm using an ELISA plate reader (LX800; Biotec, USA). Cell viability was calculated as a percentage of proliferation.

#### 3.6.4. Cell Scratch Wound Healing Assay

The migratory potential of fibroblast cells was assessed using a cell scratch wound healing assay, following a methodology described in a previous research study (22). Initially, 2 × 10^4^ cells/mL were cultured in a 48-well plate until nearly reaching confluence. Subsequently, a linear wound was created in the cell monolayer using a sterile pipette tip, and any resulting cellular debris was removed by washing the wells with PBS. Following this, CuNPs at ½ CC_50_ and CC_50_ was added and incubated for 24 hours. The cells were then examined using an inverted microscope at 0 and 24 hours. The ratio of the increase in wound closure relative to the pre-treatment value was calculated and reported as cell migration.

### 3.7. Real-time PCR for Evaluating the Expression Level of Angiogenesis and Inflammatory Genes

In order to conduct the experiment, RAW 264.7 cells were cultured at 1 × 10^5^/mL and treated with 0.1 mL lipopolysaccharide (LPS) at a concentration of 20 ng/mL, with and without CuNPs at varying concentrations (1/3 CC_50_, ½ CC_50_, and CC_50_) in a 24-well plate for a duration of 24 hours. The total RNA was isolated using a Qiagen kit, and its quality was evaluated using a Biotek Epoch nanodrop. Subsequently, cDNA synthesis was performed using a Fermentas kit, and the gene expression analysis was carried out using SYBR Green real-time PCR with specific primers for interleukin-1 (IL-1β), tumor necrosis factor alpha (TNF-α), nuclear factor kappa B (NF-κB), vascular endothelial growth factor (VEGF-A), human leukocyte antigen-G5 (HLA-G5), and the housekeeping gene GAPDH. The PCR process involved denaturation at 95°C for 10 minutes, followed by 40 extension cycles and a final cycle at 72°C for 5 minutes. The 2^⁻∆ΔCt^ method was employed with Bio-Rad iQ5 Optical System Software to determine the gene expression levels (23).

### 3.8. Statistical Analysis

The trials were conducted three times. The collected data were entered into SPSS software version 25.0 for statistical analysis. One-way analysis of variance and *t*-tests were employed to compare the data. A significance threshold of P < 0.05 was deemed statistically significant.

## 4. Results

### 4.1. The Physicochemical Structure of Green Synthesized Copper Nanoparticles

#### 4.1.1. UV-Vis Analysis

**Figure 1.**
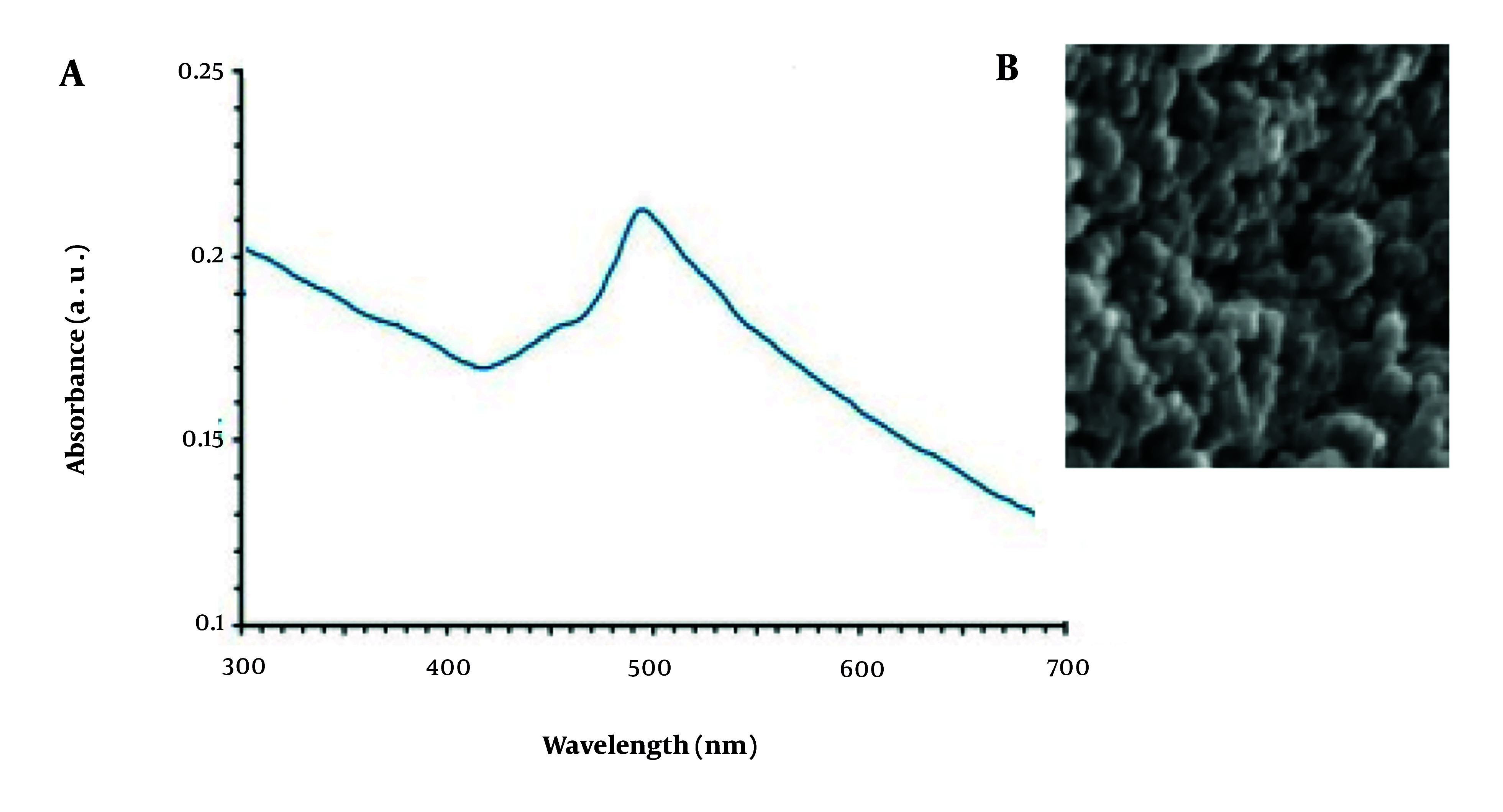
The results of the analysis of A, UV-Vis; and B, scanning electron microscope (SEM) of the obtained copper nanoparticles (CuNPs)

The UV-Vis analysis revealed an absorption peak at 491 nm for the NPs, indicating the presence of CuNPs (Figure 1A). 

#### 4.1.2. Scanning Electron Microscope Analysis

Examination using SEM revealed that the CuNPs displayed a spherical morphology, with sizes ranging from 10 to 85 nm. The majority of the size distribution was concentrated between 30 and 50 nm (Figure 1B). 

#### 4.1.3. X-ray Diffraction Analysis

**Figure 2.**
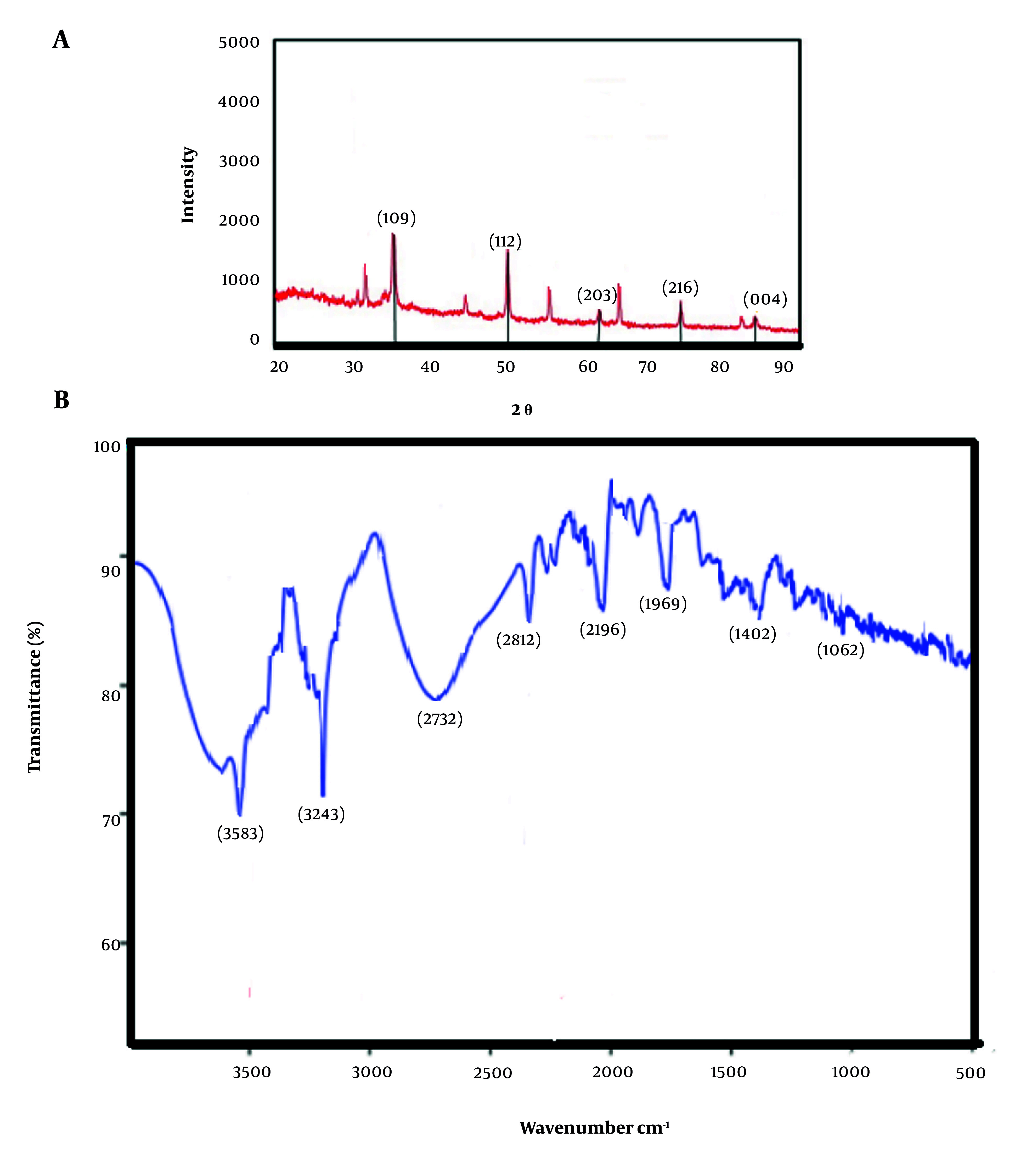
The analysis of A, X-ray diffraction (XRD); and B, the fourier transform infrared spectroscopy (FTIR) of the obtained copper nanoparticles (CuNPs)

The XRD analysis identified diffraction peaks at 38.3°, 51.2°, 62.2°, 73.1°, and 83.3° corresponding to the (109), (112), (203), (216), and (004) crystallographic planes, respectively, confirming the monoclinic crystalline phase of CuNPs (Figure 2A). 

#### 4.1.4. Fourier Transform Infrared Spectroscopy Analysis

The FTIR results indicated that the plant extract effectively reduced copper ions, suggesting its potential application as a nanoparticle coating agent. The observed bands at 3583, 3243, 2812, 2196, 1769, 1402, and 1062 cm^-1^ were attributed to various chemical interactions between the plant extract and copper ions, including O-H, C-H, C-O, and C-C for stretching of alcohol and phenol, aliphatic, ester carbonyl, aromatic ring, and C-O ester, respectively (Figure 2B). 

### 4.2. Antibacterial Effects of Copper Nanoparticles

**Figure 3.**
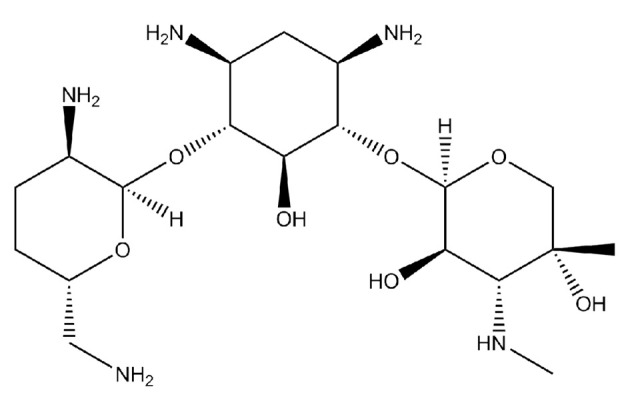
Chemical structure of gentamicin (GNT)

Table 2 presents the MIC values for CuNPs, gentamicin (GNT) (Figure 3), and GNT + cloxacillin (CLX) against the MRSA bacterial strain. The maximum antibacterial efficacy was observed for CuNPs + GNT, with MIC values of 1.33 µg/mL.

**Table 2.** Minimum Inhibitory Concentration, Minimum Bactericidal Concentration, and Minimum Biofilm Inhibition Concentration of Copper Nanoparticles, Gentamicin, and CNP + Gentamicin against *Staphylococcus aureus* Resistant to Methicillin Strain (n = 3)

Compounds	MRSA (µg/mL)		
	MIC	MBC	MBIC_50_
**CuNPs**	10.6 ± 1.15	12.0 ± 0.0	6.6 ± 2.3
**GNT**	3.3 ± 1.15	4.0 ± 0.0	1.33 ± 0.57
**CuNPs + GNT**	1.33 ± 0.57 ^a^	1.33 ± 0.57 ^a^	0.50 ± 0.0

Abbreviations: MRSA, methicillin-resistant *Staphylococcus aureus*; MIC, minimum inhibitory concentration; MBC, minimum bactericidal concentration; MBIC_50_, minimum biofilm inhibition concentration; CuNPs, copper nanoparticles; GNT, gentamicin.

^a^ P < 0.001 significant change compared with control group.

### 4.3. Effect of Copper Nanoparticles on Inhibition of Biofilm

**Figure 4.**
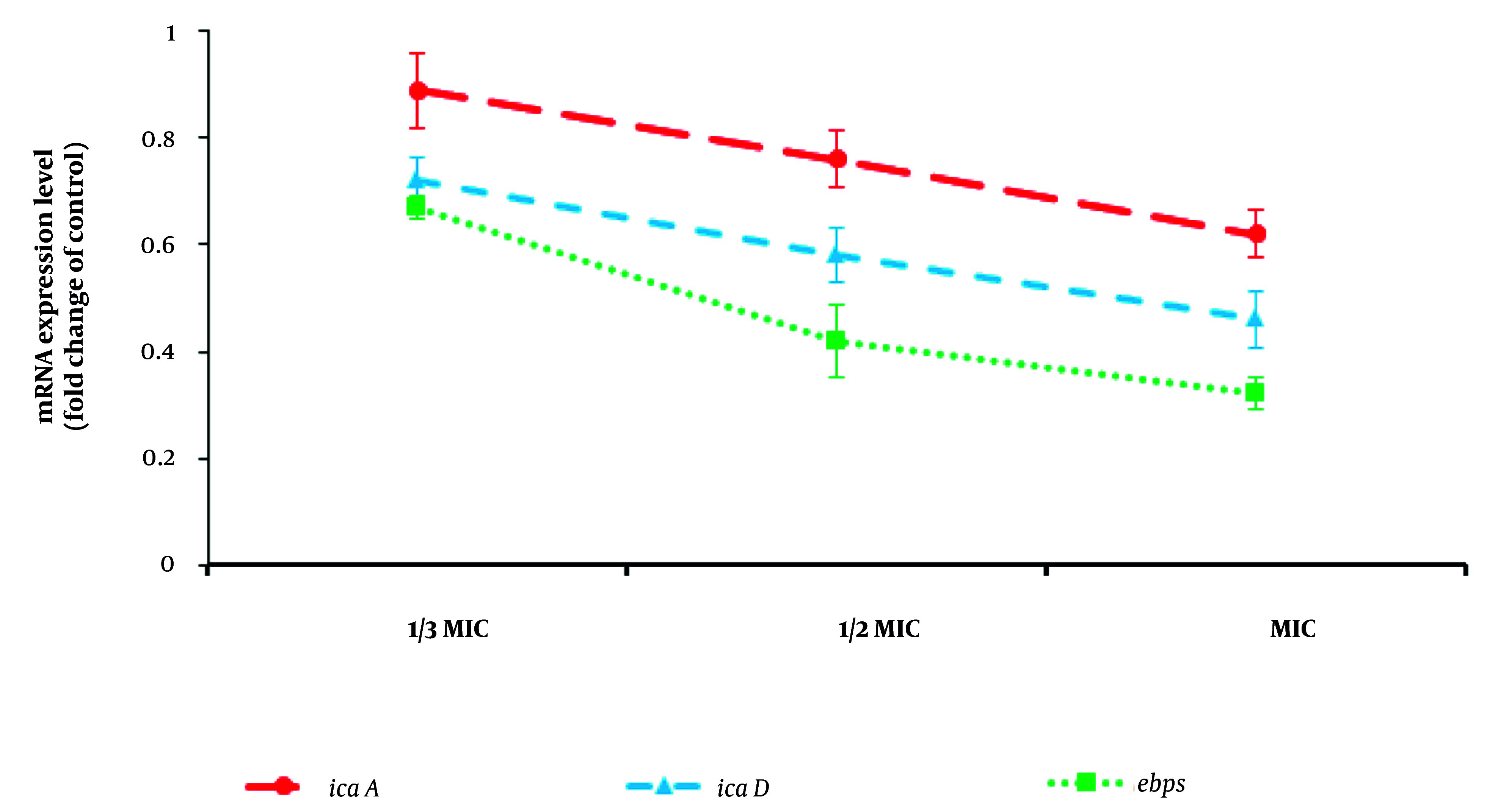
Effects of copper nanoparticles (CuNPs) on the expression level of biofilm related genes of intracellular adhesion A (icaA), intracellular adhesion D (icaD) and elastin-binding protein (EbpS) genes in *Staphylococcus aureus* resistant to methicillin strain (MRSA) (mean ± SD; n = 3)

Regarding biofilm inhibition, the findings demonstrated that CuNPs alone and in combination with GNT inhibited biofilm formation in MRSA, with minimum biofilm inhibitory concentration values of 6.6 µg/mL and 0.50 µg/mL, respectively. Additionally, Figure 4 illustrates the effect of CuNPs on the expression levels of gtf genes in the tested bacteria. The CuNPs significantly (P < 0.05) downregulated the expression levels of icaA, icaD, and EbpS in MRSA, particularly at 1/2 MIC and MIC.

### 4.4. Effect of Copper Nanoparticles on Protein Leakage

**Figure 5.**
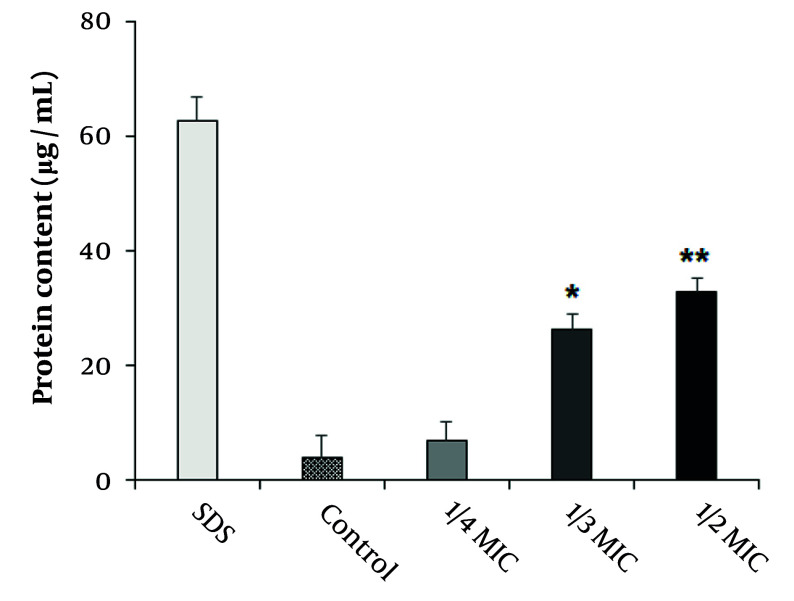
Effects of copper nanoparticles (CuNPs) on the protein leakage of *Staphylococcus aureus* resistant to methicillin strain (MRSA) at 1/4 MIC, 1/3MIC, and 1/2 MIC (mean ± SD; n = 3). * P < 0.05 and ** P < 0.01 compared with control group.

The impact of CuNPs on protein leakage in the MRSA strain is illustrated in Figure 5. The results indicated that CuNPs induced significant protein leakage at concentrations corresponding to half and one-third of the MIC in the MRSA strain (P < 0.001).

### 4.5. Wound Healing Effects of Copper Nanoparticles

**Figure 6.**
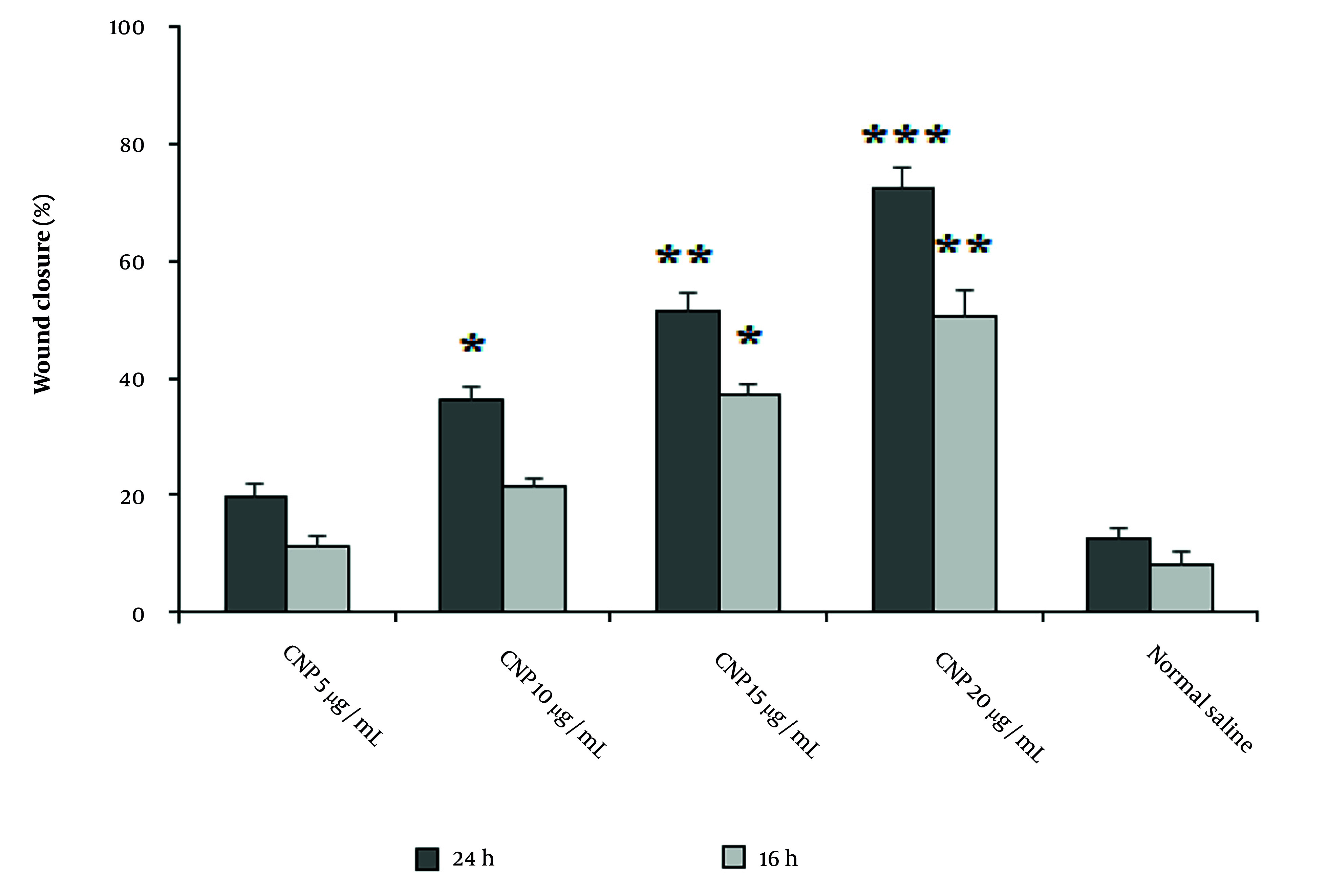
The effects of green synthesized copper nanoparticles (CuNPs) on the migration rate of scratched human skin fibroblast (Hs27), with the data presented as the mean value accompanied by the standard deviation. The * P < 0.05, ** P < 0.01, and *** P < 0.001 denotes a significant difference in comparison to the control group.

The findings from the MTT assay indicated that the determined cytotoxic concentration (CC_50_) value for CuNPs produced through eco-friendly techniques was 291.6 μg/mL. Subsequent concentrations of CuNPs selected for further analysis were based on this CC_50_ value. It was observed that these concentrations resulted in a progressive increase in fibroblast cell proliferation in a dose-dependent manner, as illustrated in Table 3. The results from the scratch test demonstrated that the application of CuNPs enhanced wound healing in a dosage-dependent manner (Figure 6). After 16 hours, the wound closure rate exhibited a dose-dependent increase with CuNPs, particularly notable at concentrations of 15 and 20 µg/mL compared to the control group. Similarly, after a 24-hour exposure, the wound closure rate showed a dose-dependent increase with CuNPs, with significant improvements at concentrations of 10, 15, and 20 µg/mL when compared to the control group.

**Table 3.** Effects of Copper Nanoparticles on Cell Viability and Proliferation in Skin Fibroblast Cells

Concentration (µg/mL)	Values
Cell cytotoxicity	
Non-treated	98.9 ± 1.05 ^a^
50 ^b^	91.4 ± 3.15 ^a^
100	82.8 ± 3.25 ^a^
200	65.7 ± 4.12 ^a^
400	31.4 ± 2.56 ^a^
**Fibroblast proliferation assay**	
5	3.14 ± 0.36 ^c^
10	9.7 ± 1.05 ^c^
15	16.2 ± 1.16 ^c^
20	29.7 ± 2.55 ^c^
-	-

^a^ Viability (%).

^b^ CC_50_ (50% of the cells) = 291.6 (µg/mL).

^c^ Proliferation (%).

### 4.6. Effect on the Expression Level of Angiogenesis Genes

**Figure 7.**
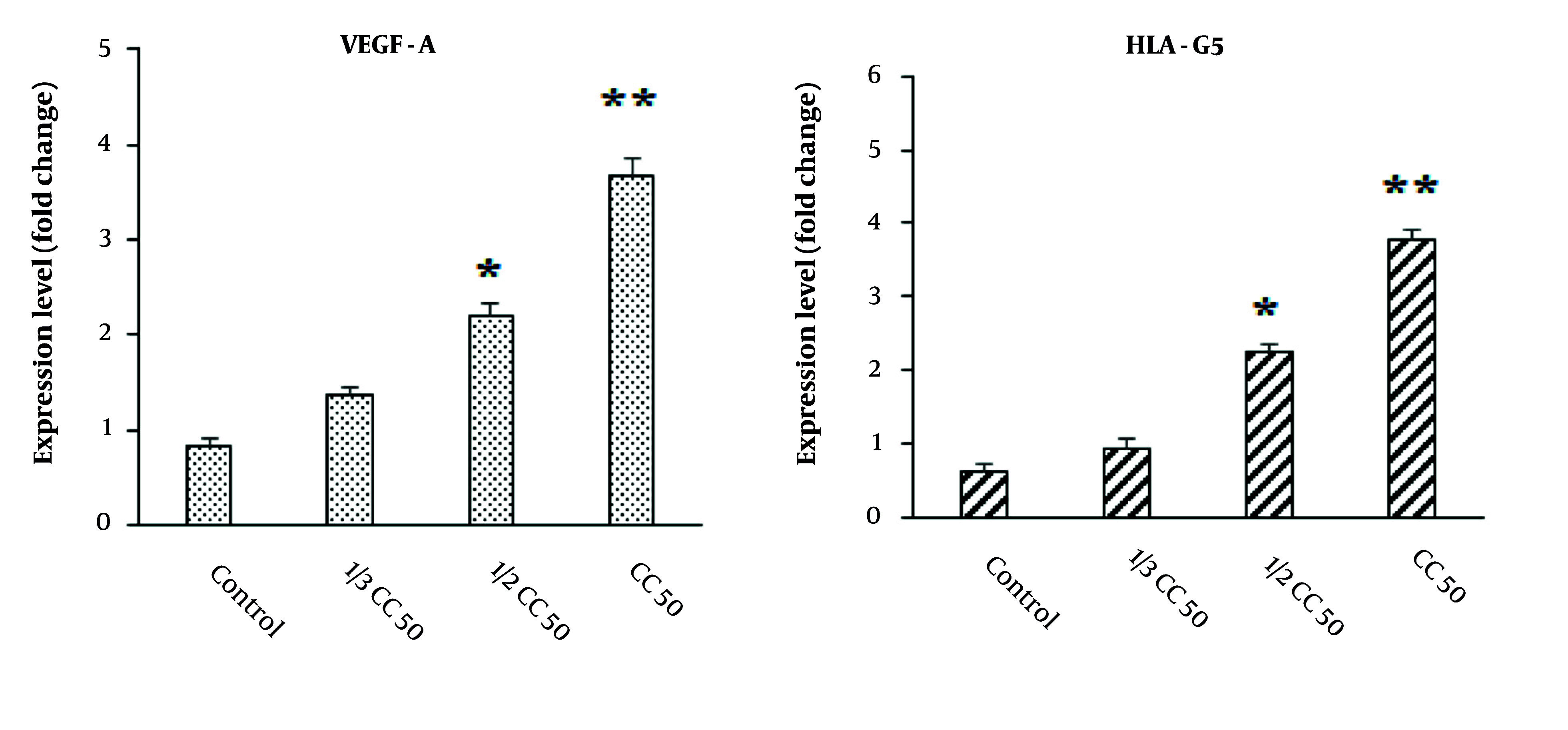
Effects of green synthesized copper nanoparticles (CuNPs) on the expression level of angiogenesis-related genes of vascular endothelial growth factor (VEGF-A) and human leukocyte antigen-G5 (HLA-G5). * P < 0.05 and ** P < 0.01 significant change compared with control group (mean ± SD; n = 3)

Utilizing real-time PCR analysis, as illustrated in Figure 7, we observed a significant upregulation in the expression of both HLA-G5 and VEGF-A genes in cells exposed to CuNPs compared to the control group (P < 0.05).

### 4.7. Effect on the Expression Level of Anti-inflammatory Genes

**Figure 8.**
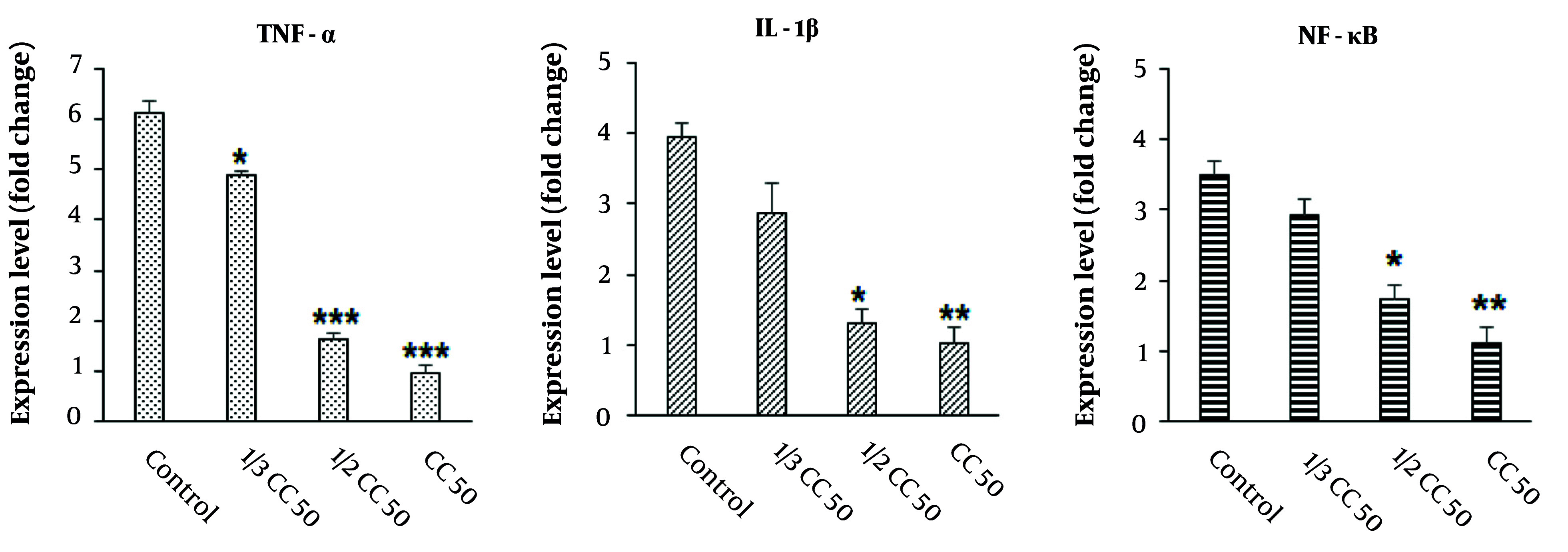
Effects of green synthesized copper nanoparticles (CuNPs) on the expression level of inflammatory-related genes of interleukin-1 (IL-1β), tumor necrosis factor alpha (TNF-α), and nuclear factor kappa B (NF-κB). * P < 0.05, ** P < 0.01 and *** P < 0.001 significant change compared with control group (mean ± SD, n = 3).

The findings from the real-time PCR analysis, as illustrated in Figure 8, demonstrated that the application of CuNPs resulted in a decrease in the expression levels of inflammatory genes, such as NF-κB, IL-1β, and TNF-α, in LPS-induced RAW 264.7 cells. This reduction was observed to be dose-dependent on CuNPs administration compared to the control group, with statistical significance (P < 0.05).

## 5. Discussion

The environmentally friendly synthesis of CuNPs represents a straightforward, cost-effective, and sustainable approach that avoids hazardous substances (24). The present study aimed to utilize a green synthesis method employing *L. arcticus* extract for the production of CuNPs, focusing on evaluating its antimicrobial, antibiofilm, and cellular mechanisms. The SEM analysis revealed that the green-synthesized CuNPs exhibited a spherical morphology, with dimensions ranging from 10 to 85 nm and a predominant size distribution between 30 and 50 nm. Previous studies have reported the green synthesis of CuNPs using plant extracts from various sources, including Ginger Lily, *Tinospora cordifolia*, *Gloriosa superba*, *Aloe vera*, and *Cordia sebestena* (24). These studies indicate that the resulting CuNPs exhibit particle sizes within the range of 5 to 130 nm (24).

By evaluating antibacterial efficacy, we found that the maximum antibacterial effect was observed for CuNPs + GNT, with MIC values of 1.33 µg/mL. Biofilms are considered a primary target for the development of antibacterial agents, as they are closely associated with the most prevalent mode of infection by microbial pathogens. Among the biofilm-related genes, icaA, icaD, and EbpS are the key genes involved in MRSA (25). Concerning biofilm inhibition, the findings demonstrated that CuNPs, both alone and in combination with GNT, inhibited biofilm formation in MRSA. Furthermore, CuNPs significantly (P < 0.05) downregulated the expression levels of icaA, icaD, and EbpS in MRSA, particularly at 1/2 MIC and MIC concentrations.

Regarding antimicrobial effects, several studies have reported the efficacy of CuNPs. For instance, Alao et al. showed that CuNPs green synthesized by ethanolic extract of *Kigelia africana* exhibited notable antibacterial efficacy against *Pseudomonas aeruginosa*, *Shigella* species, *S. aureus*, *Salmonella typhi*, and *Escherichia coli* (26). Anna Thomas et al. found that CuNPs, green synthesized using green tea at concentrations of 25, 50, and 100 µg/µL, exhibited promising antibacterial effects against various microbial isolates, including *S. mutans*, *Enterococcus faecalis*, and *Candida albicans* (27). Qamar et al., demonstrated that CuNPs synthesized using *Momordica charantia* extract was effective against multidrug-resistant clinical isolates, such as *Staphylococcus* spp. and *Klebsiella pneumoniae* (28). Additionally, Wu et al. reported that CuNPs synthesized from *Cissus vitiginea* extract significantly inhibited the growth of urinary tract bacteria, including *Enterococcus* spp., *Proteus* spp., *Klebsiella* spp., and *E. coli* (29). These differences in the results can be related to factors such as the type of bacteria, type of green synthesis, type of plant, tested concentrations, and type of antibacterial test performed.

Since proteins are considered essential factors in bacterial cells, we assessed the effects of CuNPs on protein leakage in the MRSA strain. Our findings revealed that CuNPs caused significant protein leakage (P < 0.001) at 1/2 MIC and 1/3 MIC in the MRSA strain, indicating that CuNPs indirectly affected cell membrane permeability in the MRSA strain. Prior investigations demonstrated that CuNPs exhibited antimicrobial effects against various bacteria by altering cell membrane morphology, subsequently increasing cell membrane permeability and disrupting vital metabolic processes, which ultimately lead to cell death (30). Other studies have also reported potential mechanisms of CuNPs, including the inhibition of biofilm formation, production of reactive oxygen species, protein oxidation, lipid peroxidation, and degradation of DNA (30).

The rising incidence of wounds resulting from accidents, surgeries, burns, and chronic conditions such as diabetes highlights the ongoing importance of wounds as a critical medical concern. Infections caused by pathogenic bacteria can hinder the healing process, especially when they form biofilms, which can lead to persistent infections (31). Therefore, the development of innovative materials for wound management and dressings is essential. Many nanomaterials exhibit antibacterial properties alongside wound-healing capabilities, making them potentially valuable for the treatment of various types of wounds (32). Although the wound healing effects of certain metal NPs have been reported in various studies in recent years, the findings of these studies have been questioned due to factors such as the type of NPs used, their synthesis methods, and the testing protocols (33).

In our research, we found that CuNPs exhibited significant in vitro wound healing effects through fibroblast cell proliferation and wound closure in a dose-dependent manner. In a study conducted by Hakimzadeh and Kosar, the results showed that CuNPs green synthesized by *Ferula macrecolea* extract demonstrated a dose-dependent enhancement in the rate of wound closure at both 16 and 24 hours. Furthermore, their findings from real-time PCR analysis indicated that CuNPs induced an upregulation in the expression levels of the gene responsible for inducible nitric oxide synthase (iNOS) in RAW 264.7 cells (34). The observed discrepancies in the results may be attributed to several factors, including the method of green synthesis employed, the particular plant utilized, the concentrations tested, and the nature of the wound healing assays conducted.

Vascular endothelial growth factor, a key factor in angiogenesis, acts as a stimulant for endothelial cells, a chemotactic agent, and a promoter of vascular permeability (35). Conversely, HLA-G5 plays a critical role as an immunological tolerance factor within the human body, with its expression being essential for immunomodulatory functions (35). Copper has the capacity to stimulate the expression of VEGF and extracellular matrix proteins, thereby enhancing the stability of wound healing across various wound healing mechanisms (35). Our results demonstrated a notable increase in the expression of HLA-G5 and VEGF-A genes in cells treated with CuNPs compared to the control group, indicating that CuNPs induced angiogenesis and subsequently promoted wound healing.

Numerous studies have highlighted the essential role of inflammation in the body’s defense against invading pathogens and in the removal of dead tissue at the injury site (36). However, prolonged inflammation can be detrimental, potentially disrupting the normal stages of wound healing and contributing to excessive scarring (36). Therefore, controlling and reducing inflammation can significantly enhance wound healing. Nanoparticles have been identified as promising anti-inflammatory agents in recent decades. Due to their high surface area-to-volume ratio, NPs exhibit superior efficacy in inhibiting pro-inflammatory mediators, such as cytokines and enzymes that facilitate inflammation, in comparison to their bulk material counterparts (36). Our results showed that CuNPs led to a reduction in the expression levels of inflammatory genes NF-κB, IL-1β, and TNF-α in LPS-induced RAW 264.7 cells in a dose-dependent manner, suggesting the potent role of CuNPs in controlling inflammation and subsequently improving wound healing. It has been demonstrated that green-synthesized NPs possess strong antimicrobial, antibiofilm, angiogenic, and anti-inflammatory properties, all of which contribute to enhanced wound healing.

### 5.1. Conclusions

According to the findings of this experimental test, CuNPs, in conjunction with GTM, exhibits promising antibacterial effects against MRSA without causing cytotoxicity to normal cells. The current study also demonstrated that green-synthesized CuNPs possesses significant wound-healing properties through its antibacterial activity, inhibition of biofilm formation, induction of angiogenesis, and reduction of inflammation. However, further research is necessary to clarify the precise mechanisms of action and potential toxicity of CuNPs.

## Data Availability

All data are available in publicly accessible databases under the accession numbers reported.
